# Acknowledgement to Reviewers of *Pathogens* in 2017

**DOI:** 10.3390/pathogens7010010

**Published:** 2018-01-15

**Authors:** Pathogens Editorial Office

**Affiliations:** MDPI AG, St. Alban-Anlage 66, 4052 Basel, Switzerland; pathogens@mdpi.com

Peer review is an essential part in the publication process, ensuring that *Pathogens* maintains high quality standards for its published papers. In 2017, a total of 69 papers were published in the journal. Thanks to the cooperation of our reviewers, the median time to first decision was 23 days and the median time to publication was 37 days. The editors would like to express their sincere gratitude to the following reviewers for their time and dedication in 2017:

Abdel-Whab, SayedGarner, Bianca Mohr, Ian Aboulata, A. E. Gluenz, EvaMorris, James C. Agnello, MelissaGoodhead, Ian Münch, JanAlfa, MichelleGóral, Tomasz Nachbagauer, Raffael Allen, DavidGorini Da Veiga, AnaNewburg, DavidAnastasia, VlasovaGottschalk, MarceloNissapatorn, Veeranoot Anderson, Eric A. Groveman, BradleyO’Shea, HelenAndrabi, RaieesGu, ZhenOglesby-Sherrouse, AmandaAngelici, Maria CristinaGuyard, Cyril Paeshuyse, JanAraujo, Rosa Hammarton, Tansy Paillot, R. Baird, NicholasHazlett, Linda D. Panda, AmareshBarichello, TatianaHeir, Even PARIS, Luc Bartha, IstvánHowe, Daniel K.Passler, ThomasBártová, EvaHung, NoelynPerez-Gracia, Maria-TeresaBienkiewicz, Ewa AnnaHurley, Bryan Petrova, Olga E. Bilitewski, Ursula Izumiya, YoshihirPoudel, BarunBodet, Charles Jacob, ShaleemPowell, Joshua Bouckaert, Julie Jeong, Hye Won Qin, ZhiqiangBrulois, Kevin Jones-Brando, LorraineRadecka, Hanna Bruschi, FabrizioJordan, KieranRahimion-Pour, AlexChambers, ThomasKarlsson, Erik Reese, TiffanyChen, Xian-Ming Kawano, KouichiroReichel, Michael P.Chernoff, JonathanKivistö, Rauni Schmidt, Rebecca Choi, Inyeong Kobayashi, Nobumichi Schultz, Thomas Cliffe, Anna Kodzius, RimantasSekizaki, TsutomuCox, Jonathan A.G. Koehler, AnsonSherchan, Samendra Crabtree, Jean Krause, Philip Silva, Christopher J.Cui, LongzhuKucharikova, SonaSinghrao, Sim K. Daly, Janet Kumar, Binod Sismaet, Hunter Deng, Peng Kumar, Amit Slama-Schwok, Anny Desdouits, Marion Lade, HarshadStecenko, ArleneDesselberger, Ulrich LaRock, Christopher N. Subbiah, MuruganDidonna, Alessandro Leung, Polly Supattapone, Surachai Doerig, ChristianLewis, KarenSzakal, EvelinDonnarumma, Giovanna Li, DapengTani, AkioD'Orazio, SarahLiang, Yurong Thackray, AlanaDrew, TrevorLiao, Pei-ChunTiwari, Vaibhav Ehrlich, ElanaLiu, GeorgeTretina, Kyle Engel, Daniel R.Lorenzo-Gómez, M. F. Turton, JaneEngman, David M. Lukac, DavidValero, Manuel Falkinham, JosephLuo, PeigaoVan Den Broeck, Wim Fernández, Lucía Ma, HuailiangVan Dyk, Linda F. Fiedler, TomasMadison-Antenucci, SusanVan Lint, Carine Flegr, JaroslavMahsoub, Hassan Viducic, Darija Flemington, Erik K. Margolis, ToddWhiley, Harriet Ford, Brian E.Martín, Margarita Wong, Boon-Seng Foster, Tim Maruyama, Kei Wright, James C. Fuentes, Isabel McConkey, Glenn A.Xu, GuangFukushi, HidetoMcKenzie, Debbie Yasunami, MichioGaglia, Marta MariaMenon, Vipin Zhou, WenquanGalat, AndrzejMeschwitz, Susan 


